# A Global View of Neonatal Asphyxia and Resuscitation

**DOI:** 10.3389/fped.2019.00489

**Published:** 2019-11-26

**Authors:** Robert Moshiro, Paschal Mdoe, Jeffrey M. Perlman

**Affiliations:** ^1^Department of Paediatrics and Child Health, Muhimbili National Hospital, Dar es Salaam, Tanzania; ^2^Department of Obstetrics and Gynecology, Haydom Lutheran Hospital, Manyara, Tanzania; ^3^Division of Newborn Medicine, Department of Pediatrics, Weill Cornell Medicine, New York-Presbyterian Hospital, New York, NY, United States

**Keywords:** birth asphyxia, resuscitation, apnea at birth, hypoxia-ischemia, global health

## Abstract

Birth asphyxia (BA), assumed to be related to intrapartum related hypoxia-ischemia, accounts for one million neonatal deaths annually. In the low resource setting BA is usually defined as a failure to initiate or sustain spontaneous breathing at birth. In the resource replete setting BA is a biochemical definition related to impaired gas exchange, due to interruption of placental blood flow (PBF). An umbilical arterial pH <7.00 referred to as severe fetal acidemia, reflects a degree of acidosis, where potential risk of adverse neurologic sequelae is increased. However, even with this degree of acidemia, the likelihood of mortality or adverse neurologic sequelae remains low. The aim is to focus on the definition of BA in the low resource setting, and compare it to the diagnosis in the resource replete setting, highlighting the importance of interruption of placental blood flow as it relates to morbidity and mortality. With asphyxia, the fetus aims to redistribute cardiac output to protect more vital organs e.g., brain, myocardium, and adrenal gland at the expense of decreased flow to organs such as kidney or intestine. In an experimental newborn model, animals subjected to asphyxia immediately develop primary apnea with bradycardia sustained blood pressure and normal pH. Recovery of respirations follows basic interventions, i.e. stimulation coupled with reversal of asphyxia. However, if asphyxia is sustained, secondary apnea manifests with bradycardia, hypotension, and pH <7.00. More intensive resuscitation including bag mask ventilation ± intubation ± cardio-pulmonary resuscitation may be necessary for correction upon reversal of asphyxia. Identification of a severely acidemic state (cord arterial pH < 7.00) in the newborn, may help to differentiate the truly asphyxiated intrapartum related cases that result in mortality, from those cases where mortality is related to delay in or ineffective basic resuscitation.

## Background

It is estimated that globally 2.5 million newborn deaths occur annually contributing to ~47% of the under-5 child mortality (1, 2). Birth asphyxia (BA), assumed to be related to intrapartum hypoxia- ischemia, accounts anywhere from 30 to 35 percent of neonatal deaths (3). This translates into an estimated one million neonates who die each year worldwide (1–3). In addition, an estimated 1.3 million newborns are reported to be “fresh stillborn” (FSB) suggestive of an intrapartum demise, shortly before delivery (4, 5) The first day and especially the first hour is critical to newborn survival with the highest risk of intrapartum-related neonatal deaths (60–70%), occurring within 24 h of birth (1–3). This review will focus on the definition of BA in the low resource setting and compare it to the diagnosis in the resource replete setting, highlighting the importance of interruption of placental blood flow. In the resource limited setting, identification of a severely acidemic state may help to differentiate the truly intrapartum related BA cases resulting in mortality, from those cases where mortality is related to delayed or ineffective basic resuscitation.

## Defining Birth Asphyxia

BA in the low resource setting is usually defined as a failure to initiate or sustain spontaneous breathing at birth and in some circumstances includes a 1-min Apgar score <7.00 (3). Contrast this with the resource replete setting where BA is a biochemical definition, related to impaired gas exchange, due to interruption of placental blood flow (PBF), with progressive hypoxemia, hypercapnia, and acidosis. BA is identified by the presence of fetal acidosis in cord umbilical arterial blood following delivery of the baby. However, the umbilical arterial pH that best defines this state remains unclear. Traditionally, asphyxia was defined as a cord umbilical arterial pH <7.20 (6). Using this definition, the risk for death or neurodevelopmental sequelae was exceedingly low (7). It has long been accepted that an umbilical arterial pH <7.00 reflects a degree of acidosis, often referred to as severe fetal acidemia, where the risk of adverse neurologic sequelae is increased (8, 9). A cord pH <7.00 complicates ~0.3% of all deliveries, i.e., 3 per 1,000 term deliveries (8). However, even with severe fetal acidemia, the likelihood of subsequent brain injury or mortality is low. Moreover, it has been shown that most infants, i.e., >60 percent with a cord pH <7.00 have a normal labor and delivery course, initiate breathing shortly after delivery, are triaged to the regular nursery, and are discharged home within 24 h (10). Even those infants with severe fetal acidemia admitted to intensive care (often because of respiratory difficulties) exhibit a benign neurologic course in most cases. Only a small percentage present with moderate to severe encephalopathy, with adverse outcome, i.e., either death or subsequent cerebral palsy (11–13). This observation points to the intrinsic resistance of the brain to severe asphyxia.

## Physiology of Asphyxia

### Circulatory Responses to Interruption of Placental Blood Flow to Preserve Cerebral Blood Flow

When placental blood flow (PBF) is compromised, the fetus redistributes cardiac output to protect vital organs such as brain, heart, and adrenal gland at the expense of flow to the kidney, intestine and skin. Several factors contribute to this response including hypoxemia which induces pulmonary vasoconstriction. This results in reduced pulmonary blood flow, left atrial blood return, and a decrease in left atrial pressure (14, 15). There is an increase in right-to-left shunting across the foramen ovale, resulting in delivery of more oxygenated blood to the left heart which is preferentially directed to the brain and heart. Within the brain, hypoxemia results in a decrease in cerebral vascular resistance. In experimental studies it has been shown that this resistance can fall by as much as 50%, resulting in an increase in cerebral blood flow. This compensates for the decreased blood oxygen content observed during the initial phase of asphyxia. When the asphyxial process is prolonged and/or severe, systemic blood pressure falls to a point where compensatory mechanisms fail and circulatory collapse ensues. This critical threshold varies amongst fetuses, and represents a point below which the cerebral circulation can no longer dilate to maintain flow (16–18). At this juncture, cerebral oxygen delivery is insufficient to meet cellular demand, and brain injury occurs.

### Respiratory Responses to Asphyxia

**Figure 1:**
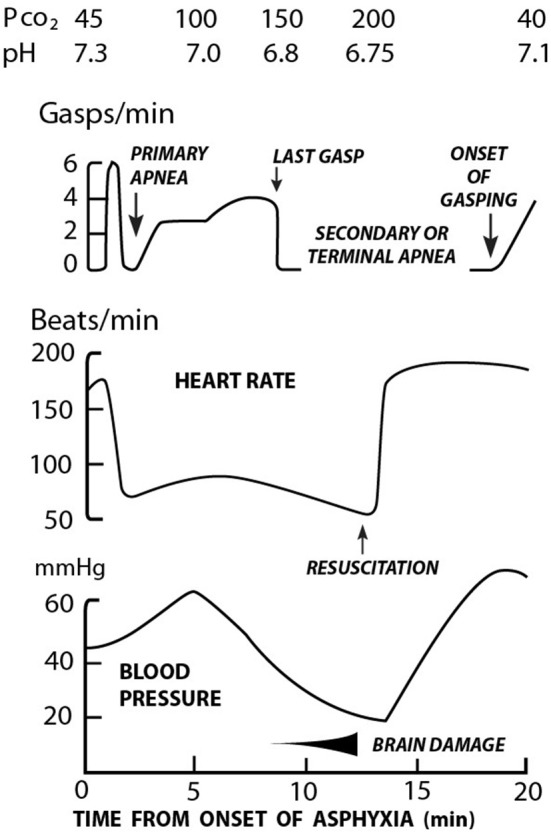
Relationship between respiration, heart rate, blood pressure, and acidosis in rhesus monkeys during asphyxia and resuscitation [Adapted from Dawes et al. (19)].

In addition to the cardiovascular changes described above associated with asphyxia, characteristic changes in the breathing patterns occur. These cardio-respiratory changes with asphyxia have been elegantly described by Dawes et al. (19) (Figure 1). Using the rhesus monkey, asphyxia was initiated by ligating the umbilical cord and covering the head with a small bag of warm saline. A characteristic series of changes were immediately observed. Within 30 s a brief period of rapid rhythmic respiratory effort occurred. This culminated in apnea (primary) and bradycardia (heart rate (HR) >60 beats/min (bpm) in most cases. If the asphyxial process was interrupted (usually within 30 to 90 s), the heart rate invariably responded to basic interventions including drying, stimulation and/or bag mask ventilation, if implemented in a timely manner (19). If the asphyxial process was allowed to continue, the animal then began to gasp. If the asphyxial process was interrupted spontaneous regular respirations could still be induced via prompt physical stimulation. Without intervention, gasping lasted for ~4 min, gradually became weaker until a terminal “last gasp” occurred. This was deemed secondary apnea with a HR invariably <60 bpm and the pH <7.00. Without resuscitation, death followed. Notably for every 1 min delay in initiating resuscitation at this stage of asphyxia, it took ~2 min before gasping was observed, and 4 min until respirations became apparent (19).

### Transitional Circulation Changes at Birth

With transition from *in utero* to *ex-utero* life, significant circulatory changes occur closely intertwined with concurrent respiratory changes. Upon crying at birth, the lungs rapidly expand and pulmonary vascular resistance drops. The HR correspondingly increases rapidly from around 120 to 150 bpm within 10 to 25 s, following the onset of breathing (20). This results in a significant increase in pulmonary blood flow, a decrease and then reversal of right-to-left ductal shunting as pulmonary artery pressure falls below systemic blood pressure. There is an increase in pulmonary venous return to left atrium, with an increase in pressure which exceeds right atrial pressure. This causes the foramen ovale to functionally close. Following umbilical cord clamping and removal of the low resistance placenta, systemic vascular resistance increases with a concomitant increase in systemic blood pressure.

### Impact of Interruption of PBF on Cardio-Respiratory Adaptation

With a cord arterial pH <7.00 the infant may be delivered flaccid with no to minimal respiratory effort, absent lung expansion, continued elevated pulmonary pressure, and an increase in systematic vascular resistance upon placental separation. Hypoxia, hypercarbia and acidosis will rapidly worsen, unless resuscitation efforts are immediately initiated. Implementation of the basic intervention steps as outlined in the Helping Babies Breathe (HBB) algorithm (21), followed by effective positive pressure ventilation (PPV), results in lung expansion with reversal of respiratory acidosis in most cases, and the onset of spontaneous respirations.

### Other Causes of Interrupted Cardio-Respiratory Adaptation

Importantly, the diagnosis of BA based on the absence of spontaneous respirations at birth may not reflect or be related to interruption of PBF (see above). Delayed onset of breathing may be related to many causes including maternal conditions such as fever (22) or maternal medications, neonatal factors such as immaturity or anomalies. These infants are likely to be in a state of primary apnea and respond to drying, maintaining warmth, and stimulation with the onset of spontaneously breathing. If resuscitation is delayed and PPV is not instituted in a timely manner, hypoxia, hypercarbia, and acidosis can rapidly evolve and the infant can progress to an “asphyxiated state” (pH <7.00) or even to a “FSB.”

### Causes of Perinatal Asphyxia—Importance of Interruption of PBF

During labor interruption of PBF leads to BA. Two elements are critical in this regard, namely duration and the severity of the interruption. A decrease in placental blood flow may be secondary to maternal conditions such as hypertension or preeclampsia secondary to altered placental vasculature. Maternal hypotension secondary to a medication effect or spinal anesthesia may compromise PBF. Indeed, in one report spinal anesthesia was the commonest cause of an unanticipated cord arterial pH <7.00 likely related to maternal hypotension and reduced PBF (10). Placental conditions such as abruption and feto-maternal hemorrhage may also compromise PBF. Chorioamnionitis and funisitis have been linked to placental compromise and asphyxia (10). Finally, the umbilical cord may be compressed extrinsically, with conditions such as a nuchal cord or cord prolapse. In summary, methods to consistently detect fetal heart rate (FHR) abnormalities, is a critical first step to identifying the high-risk fetus, and could be greatly enhanced by continuous FHR monitoring.

### Reconciling the Global Differences in BA

Several factors may play a role in explaining this striking disparity in diagnosis as well as outcome (brain injury or mortality) with BA. First, as noted above, absence of respiratory effort at birth may manifest in the presence or absence of a cord pH <7.00. At a physiologic or clinical level, the question is whether the infant is in primary or secondary apnea (19). This in part can often be identified by events that occur during labor, i.e., FHR abnormalities (both duration and severity are important) or acute events, e.g., abruption placentae. Indeed, as a fundamental premise, we presume that most infants are in primary apnea, and will respond to the basic steps in resuscitation (dry, provide warmth, and stimulate) with the onset of spontaneous respirations. This was the primary hypothesis following the launch of the HBB training program in Tanzania. Indeed a 47% reduction in 24-h neonatal deaths was demonstrated (21). In addition a 24% reduction in FSB rates was noted (21). The significant reduction in BA related mortality and FSB, appeared in part due to the increased frequency of stimulation provided at birth (from 47 to 87%), with the subsequent initiation of spontaneous breathing. Furthermore, a concomitant significant reduction in the need for PPV was noted. Interesting in that report, of the infants who died, there was no difference in the amount of stimulation provided pre vs. post HBB training, i.e., 93 and 97%, as well as the provision of PPV, i.e., 87 vs. 97%, respectively (21).

Second, it has also been previously demonstrated in the low resource setting, that time to initiation, as well as the duration of PPV, is significantly longer among infants who died compared to infants with normal outcomes. Specifically, the risk for death increased 16 percent for every 30 s delay in initiating PPV up to 6 min, and 6 percent for every minute of applied BMV (23). Third, the inability to administer effective and sustained PPV, due to frequent interruptions in PPV delivery, to establish an ineffective mask seal, and/or an inability to intubate in many settings, are likely additional contributing factors.

A clinical study that examined the relationship between the presence of severe fetal acidemia, onset of breathing in the delivery room, and subsequent neurologic state supports the above observations (24). The clinical findings of two groups delivered in the presence of severe fetal acidemia (umbilical arterial pH ≤ 7.00, base deficit ≥12 mEq/l) are described. The first, Group I (*n* = 17), required minimal delivery room (DR) resuscitation, did not require respiratory support, and had a normal neurologic outcome. The second, Group II (*n* = 7), all required intubation and mechanical ventilation in the DR. All seven infants evolved to the syndrome of hypoxic–ischemic encephalopathy (HIE) including seizures, which developed within the first 6–12 h after birth. The cord umbilical arterial blood gases were notable for the following: first, a lower cord pH, i.e., 6.75 ± 0.18 vs. 6.90 ± 0.09 (*P* < 0.005) and a higher cord PaCO_2_ higher, i.e., 141 ± 37 vs. 94 ± 22 mmHg (*P* < 0.005) in group II vs. group I infants, respectively. The mean initial postnatal pH was lower, i.e., 7.06 ± 0.15 vs. 7.25 ± 0.09 and the PaCO_2_ remained higher in group II vs. group 1 infants, i.e., 45 ± 14 mmHg, and 30 ± 6 mmHg, respectively (*P* < 0.005). These observations point to the basic difficulty in defining BA based on the absence of respiratory effort at birth, particularly without a cord arterial blood sample. Absence of respiratory effort with a normal cord arterial pH would suggest primary apnea (Figure 1). Death under these circumstances likely reflects delayed or ineffective ventilation (see above). Conversely absent respiratory effort with severe fetal acidemia suggests secondary apnea with an increased risk for mortality. Delay in initiating or providing ineffective respiratory support will rapidly exacerbate the already acidemic state and lead to rapid demise. Finally, normal respiratory adaptation at birth may occur with severe fetal acidemia (see above) These observations may in part explain the striking differences in mortality related to BA between low and high resource settings.

### Identifying the Infant at High Risk for Interruption of Placental Blood Flow During Labor

Intermittent auscultation with the fetal stethoscope has largely been the primary method of fetal monitoring available in many low resource settings. The recent development of a novel strap-on continuous FHR monitor, called Moyo (Laerdal Global), has facilitated a more rapid identification of an abnormal FHR, and may be a breakthrough in identifying fetuses at high-risk of intrapartum hypoxia-ischemia. In a recent study in a low risk population, it was shown that implementation of continuous FHR monitoring using the Moyo device, was associated with a 6.90-fold increased detection of an abnormal FHR, i.e., absent, FHR <120 or FHR>160 bpm, and a shorter time interval from the last FHR assessment to delivery (25). A cesarean section (CS) delivery was 5.7-fold, and a vacuum extraction delivery 3.8-fold more likely following the introduction of Moyo, as a result of detection of an abnormal FHR. Overall, the need for resuscitation interventions was less post-implementation. Perinatal outcomes, i.e., fresh stillbirths and early neonatal deaths, were similar between time periods, likely reflecting a low occurrence of these morbidities in this low-risk population. While the continuous Doppler technique detects FHR abnormalities more readily than with the intermittent auscultation method, the time to a CS delivery was not different between the monitoring groups (26). This implies that it is not only the timely ability to detect fetal jeopardy during labor, but resources, i.e., insufficient operating room capacity and competent providers, must be readily available. Additionally, it is essential that one weighs the advantages of continuous FHR monitoring (early detection of FHR abnormalities, simultaneous management of several patients) versus the disadvantages (more CS deliveries) in the absence of neonatal benefit. Since the patient populations studied in these reports were low risk, future randomized studies in high risk patients are essential

Finally it would be useful to have the ability to measure cord arterial umbilical blood gases following a delivery and/or or an early postnatal pH and base deficit, to provide objective measurement of the acid base status, and by default the extent of interruption of PBF around the time of birth.

## Resuscitation in the Delivery Room

The overwhelming majority of newborns (85%) will initiate spontaneously respiratory effort within 15 s following birth, an additional 10 percent will respond to stimulation and/or suctioning (23). Another 3 to 5 percent will require PPV ± intubation and only 0.1 percent (1 in 1,000 infants) will require cardiopulmonary resuscitation (CPR), chest compressions ± medications e.g., epinephrine (27). The latter point stresses the importance of effective PPV to recovery of spontaneous circulation. Notably the HBB program does not include provision of CPR (21, 28).

The key approach to resuscitation irrespective of the setting is anticipation of a high-risk infant e.g., FHR abnormalities, prolapsed cord, prematurity. Where possible there should be two providers at the delivery capable of initiating basic stabilization/resuscitation in a timely manner. The first step should be a prebrief, where the possibility of receiving a depressed baby, and preparation for initiation of HBB or the neonatal resuscitation program (NRP) guidelines, including implementation of PPV should be discussed (28, 29). As noted above, most babies are in primary apnea and will respond to drying, providing warmth and stimulation with the onset of spontaneous breathing. Implementation of early PPV should facilitate correction of respiratory acidosis and the onset of spontaneously respirations, in the majority of infants who remain depressed. There are two important points to note. First, the early determination of heart rate is a critical marker of effective ventilation with a rapid increase in rate noted within 30 s. Second, for those non breathing newborns with persistent bradycardia (HR <100 bpm), correction steps as outlined by the mnemonic MRSOPA (29) (see Table 1) or the ability to intubate (where available), and provide PPV should result in more effective ventilation.

**Table 1:** Mnemonic used to remind providers of optimizing ventilation.

	** ACTIONS**
M	Adjust **Mask** to assure good seal on the face
R	**Reposition** airway by adjusting head to “sniffing” position
S	**Suction** mouth and nose of secretions, if present
O	**Open** mouth slightly and move jaw forward
P	Increase **Pressure** to achieve chest rise
A	Consider **Airway** alternative (endotracheal intubation or laryngeal mask airway)

The potential pathways to BA related mortality in term infants in the low resource setting was recently described (30). The majority of infants (92%) required PPV in the DR and the severity of the respiratory compromise was evident by the fact that the majority of infants who died had severe hypoxia (saturation <80%) as well as moderate to severe hypothermia (temperature <36°C) noted upon admission to the neonatal ward. Moreover, these newborns were much more likely to evolve to moderate to severe encephalopathy with seizures noted in 30% of cases. Even without evidence of severe fetal acidosis, the constellation of these clinical findings is strongly suggestive of BA as the pathway to early death in most of these infants.

## Implications

Despite the difficulties in defining BA in resource limited settings, potential opportunities for reducing BA related mortality exist. More than half of newborns who died of BA had a normal FHR during labor intermittently determined using a fetoscope. Continuous FHR monitoring may facilitate identification of such infants, and expedite delivery of fetuses in jeopardy (25). Post-delivery, more effective resuscitation including early and continuous PPV, maintaining a seal, CPAP and/or intubation (where available), minimizing hypoxia, and maintaining normal temperature are strategies to potentially reduce mortality (28). Additional basic steps including available equipment, trained staff, a skilled person at every birth are essential. Finally identification of a severely acidemic state in the newborn, may help to differentiate the truly asphyxiated intrapartum related cases resulting in mortality, from those cases where mortality is related to delayed or ineffective basic resuscitation.

## Author Contributions

All authors listed have made a substantial, direct and intellectual contribution to the work, and approved it for publication.

### Conflict of Interest

The authors declare that the research was conducted in the absence of any commercial or financial relationships that could be construed as a potential conflict of interest. The reviewer SN declared a past co-authorship with one of the authors JP to the handling editor.
